# Relationship of C-reactive Protein/Serum Albumin Ratio and qPitt Bacteremia Score With An All-Cause In-Hospital Mortality in Patients With Bloodstream Infections

**DOI:** 10.7759/cureus.66584

**Published:** 2024-08-10

**Authors:** Eyad J Al Shaqri, Abdullah Balkhair

**Affiliations:** 1 College of Medicine and Health Sciences, Sultan Qaboos University, Muscat, OMN; 2 Infectious Disease, Sultan Qaboos University Hospital, Muscat, OMN

**Keywords:** mortality, quick pitt bacteremia score, c-reactive protein/albumin ratio, sepsis, bloodstream infection

## Abstract

Background: Bloodstream infections remain a major cause of morbidity and mortality despite notable advances in their diagnosis and treatment. C-reactive protein/serum albumin ratio and the quick Pitt bacteremia score are two useful tools for clinicians to assess severity and predict mortality risk in patients with sepsis attributable to bloodstream infections. This study examined the relationship between C-reactive protein/serum albumin ratio and Q Pitt bacteremia score with all-cause in-hospital mortality in patients with bloodstream infections.

Methods: Hospitalized adult patients with bacteremic bloodstream infections between January 1, 2020, and December 31, 2021, were retrospectively reviewed. Patients’ demographics and clinical and laboratory data were retrieved from patient electronic records. C-reactive protein/albumin ratio was calculated using CRP (mg/L) and serum albumin (g/L) values obtained within 24 hours of blood culture collection and quick Pitt bacteremia score was calculated for each patient with each of the five variables of the score determined within 24 hours of blood culture collection and each patient was assigned a numerical score of 0-5 accordingly. The relationship between C-reactive protein/albumin ratio and quick Pitt bacteremia score with all-cause in-hospital mortality was determined.

Results: A total of 187 hospitalized adult patients with non-repeat bacteremic bloodstream infections were identified.* Escherichia coli* was the most common Gram-negative blood isolate while *Staphylococcus aureus* was the predominant Gram-positive isolate. One hundred and five (56.1%) patients were male with a cohort mean age of 56.9 ± 2.7 years. All-cause in-hospital mortality was 27.3%. The mean CRP/albumin ratio (8.6 ±1.7) and mean quick Pitt bacteremia score (2.8 ±0.4) were significantly higher in patients with bloodstream infections who died during their hospitalization compared to those who survived. The all-cause in-hospital mortality was 8%, 12%, 22%, 46%, 93%, and 100% for patients with quick Pitt scores of 0, 1, 2, 3, 4, and 5, respectively.

Conclusion: In hospitalized patients with bacteremic bloodstream infections, an incremental increase in quick Pitt bacteremia score and mean C-reactive protein/albumin ratio of >8 was associated with higher mortality.

## Introduction

Bloodstream infection (BSI) is defined as a positive blood culture in a patient with systemic signs of infection and may be either primary (without identified source or origin) or secondary to a documented source [1]. Despite great advances in medical diagnosis and therapeutics for sepsis including BSI, BSI remains a major cause of infectious disease morbidity and mortality and is the seventh most common cause of death [2]. Moreover, BSI can lead to sepsis, an extreme systemic response to infection, which is associated with increased mortality, prolonged length of hospital stay, and additional medical costs [3]. Mortality associated with BSI ranges from 14% for community-onset BSI to 30% for patients with severe comorbidities, such as cirrhosis, onco-hematologic diseases, or solid-organ transplants [4]. Accurate identification of predictors associated with mortality in patients with BSI is critical to informing clinical interventions and improving clinical outcomes [5]. Hence, a fast, easy, and accessible tool is required to predict mortality in patients with BSI.

C-reactive protein (CRP) and serum albumin have been shown to have utility in predicting outcomes in patients with sepsis including those with BSI [6]. CRP was discovered in 1930 by Tillett and Francis [7]. CRP is an acute-phase protein produced by the liver [8]. CRP level rises primarily in response to the interleukin-6 (IL-6) action on the CRP transcription gene which is activated in the acute phase of inflammation [7]. CRP helps the body to recognize foreign pathogens and clear them along with damaged cells [7].

Albumin is one of the most abundant blood plasma proteins. Serum albumin transports various compounds through the bloodstream such as hormones, fatty acids, etc. Furthermore, serum albumin contributes to maintaining osmotic blood pressure [9]. In critical care, albumin acts as a negative acute-phase reactant, and the level of hypoalbuminemia is associated with the severity of inflammation induced by infection [10].

It is being postulated that CRP and serum albumin are useful markers that can predict mortality in patients with sepsis [11]. Furthermore, high CRP and low serum albumin levels are associated with high mortality and poor prognosis [6,11]. Two studies by Oh et al [11] and Park et al [6] examined the relationship between the CRP/albumin ratio and the risk of 30-day mortality. Both studies showed that a higher CRP/albumin ratio was associated with increased mortality in critically ill patients [6,11].

Similarly, several clinical scoring systems have been used to assess the severity of BSI such as Pitt and Q Pitt bacteremia scores [6]. Pittsburgh University developed the Pitt bacteremia score which has been used to determine the severity of acute illness and predicts mortality in patients with BSI and has been in clinical use for nearly 30 years [12]. Pitt bacteremia score has been applied to classify patients with BSI according to the acute severity of their illness [13]. Several studies have shown that Pitt’s bacteremia score outperforms other acute severity of illness scores in different clinical settings [13,14].

Quick Pitt bacteremia score (qPitt) was recently derived from the Pitt bacteremia score as a quick version of it using 5 binary variables [12]. Patients with BSI can be stratified by qPitt according to the severity of acute illness. qPitt is a simple score that can be calculated by using initial patient evaluation with the inclusion of the following parameters: blood pressure, temperature, mental status, cardiac arrest, and the absence or presence of mechanical ventilation [13]. Patients with qPitt of 4 or more are considered critically ill with higher mortality risk [14].

This study examined the relationship between C-reactive protein/serum albumin ratio and qPitt bacteremia score and all-cause in-hospital mortality in patients with BSIs admitted at an academic, referral hospital in Oman.

## Materials and methods

Study design

This is a retrospective study. Hospitalized adult patients (age ≥18 years) with a diagnosis of bacteremic BSI and clinical signs and symptoms of sepsis confirmed by positive blood culture for pathogenic (non-contaminant) bacterial isolate during the period from January 1, 2020, to December 31, 2021, were included. Pediatrics (defined for the purpose of the study as age <18 years), non-hospitalized patients, and patients with candidemia were excluded.

Data collection and definitions

Data were obtained using the hospital’s electronic patient records (InterSystems TrakCare®) and include demographics and clinical parameters such as sex, age, date of admission, date of collection of positive blood culture, and admission to the intensive care unit. Similarly, laboratory parameters including blood culture isolates, CRP, and serum albumin were gathered within 24 hours of blood culture collection. Components of qPitt (blood pressure, temperature, mental status, cardiac arrest, and the absence or presence of mechanical ventilation) were collected. Additionally, early warning scores (EWS) extracted from vital signs records were totaled for each patient. qPitt was calculated for each patient within 24 hours of blood culture collection with the worst reading on the calendar day of the index blood culture was recorded. Each of the five variables of the score (temperature, blood pressure, mental status, cardiac arrest, presence, or absence of mechanical ventilation) was categorized into normal (0) and abnormal (1), and each patient was assigned a numerical score of 0-5 accordingly. CRP/albumin ratio was calculated using CRP (mg/L) and serum albumin (g/L) values within 24 hours of blood culture collection. The relation between qPitt, CRP/albumin ratio, and all-cause in-hospital mortality was determined.

Statistical analysis

Quantitative variables were expressed as mean ± standard deviation or median and were compared using a two-sample t-test or Mann-Whitney test, depending on whether they were normally distributed or not. Qualitative variables were expressed as percentages and compared using the Chi-square test. The significance level for statistical testing was defined as two-tailed p < 0.05. All statistical analyses were performed using the Statistical Package for the Social Sciences (SPSS).

Ethical approval

The study protocol was approved by the medical research and ethics committee of the College of Medicine and Health Sciences, Sultan Qaboos University, Oman (MREC#2745). All data was anonymized. Informed consent was not required.

## Results

A total of 187 hospitalized adult patients with non-repeat bacteremic BSI events fulfilling the inclusion criteria were identified during the study period. 105 (56.1%) of patients were male and the remaining (43.9%) were female. The mean age of the cohort was 56.9 ± 2.7 years. 124 (66.3%) of the blood culture bacterial isolates were Gram-negative-bacilli while 63 (33.7%) of the isolates were Gram-positive cocci. *Escherichia coli* was the most common Gram-negative blood isolate while *Staphylococcus aureus* was the predominant Gram-positive isolate (Table 1).

**Table 1:** Blood culture bacterial isolates from 187 patients with bloodstream infections. *ESBL: extended spectrum beta lactamase; ^MDRA: multidrug-resistant *Acinetobacter baumannii*; MRSA~: methicillin-resistant *Staphylococcus aureus*; VRE+: vancomycin-resistant Enterococcus.

	N	%
Gram-negative isolates	124	66.3
Escherichia coli	75 (ESBL*=27)	40.1
Klebsiella pneumoniae	27 (ESBL=14)	14.4
Pseudomonas aeruginosa	12	6.4
Acinetobacter baumannii	7 (MDRA^^^=1)	3.7
Klebsiella aerogenes	3	1.6
Gram-positive isolates	63	33.7
Staphylococcus aureus	35 (MRSA^~^=12)	18.7
*Enterococcus faecalis*/faecium	22 (VRE^+^=2)	11.8
Streptococcus pneumoniae	4	2.1
Streptococcus pyogenes	2	1.1
Total blood culture isolates	187	100

Fifty-one of the 187 patients with BSI died during their hospitalization resulting in an all-cause in-hospital mortality of 27.3%. Patients with BSI who died during their hospitalization were significantly older than patients who were alive at the time of discharge (62.6 ± 3.9 years vs. 54.8 ± 3.3 years, respectively). (p < 0.05). Mortality rates were similar between male and female patients (26.7% and 28.0%, correspondingly).

Mean CRP (199 ±39 mg/L), mean serum albumin (25 ±2 g/L), and the mean CRP/albumin ratio (8.6 ±1.7) were all significantly higher (p < 0.05) in patients with BSI who died during their hospitalization compared to patients who survived. Similarly, a higher mean EWS (10.2 ±0.9) and a higher qPitt bacteremia score (2.8 ±0.4) were both statistically associated with all-cause in-hospital mortality in patients with BSI (Table 2).

**Table 2:** Baseline characteristics of 187 patients with BSI stratified by all-cause in-hospital mortality. Note: Data on CRP was available for 179 patients (missing for 4 patients from each group). Data on serum albumin was available for 164 patients (missing for 18 and 5 patients from the alive and died groups respectively). Data on both CRP and serum albumin was concurrently available for 161 patients (missing for 21 and 5 patients from alive and died groups, respectively). BSI: bloodstream infection; CRP: C-reactive protein; EWS: Early Warning Score

Characteristic	Alive Mean or n (%)	Died Mean or n (%)	Total Mean or n (%)
Patients with BSI	136 (72.7%)	51 (27.3%)	187
Age (years)	54.8 ± 3.3	62.6 ± 3.9	56.9 ± 2.7
Male	77 (73.3%)	28 (26.7%)	105
Female	59 (72.0%)	23 (28.0%)	82
Admission to ICU	6 (33%)	12 (67%)	18 (9.6%)
CRP (mg/L)	153 ±21	199 ±39	166 ±19
Albumin (g/L)	32 ±1	25 ±2	30 ±1
CRP/serum albumin ratio	5.3 ±0.8	8.6 ±1.7	6.2 ±0.8
EWS	6.2 ±0.5	10.2 ±0.9	7.3 ±0.5
Q Pitt bacteremia score	1.2 ±0.2	2.8 ±0.4	1.6 ±0.2

Of the 187 evaluable patients, 49 (26%) had a qPitt bacteremia score of 0, 41 (22%) had a score of 1, 51 (27%) had a score of 2, 26 (14%) had a score of 3, 15 (8%) had a score of 4, and 5 patients (3%) had a Q Pitt score of 5. Of the 51 patients who died in hospital, 31 (61%) had a qPitt score of 3 or more. Contrarily, 15 patients (11%) with a qPitt score of 3 or more survived. The all-cause in-hospital mortality was 8%, 12%, 22%, 46%, 93%, and 100% for patients with qPitt scores of 0, 1, 2, 3, 4, and 5, respectively (p value=.05) (Table 3).

**Table 3:** Q Pitt bacteremia score and mortality stratified by score category.

Q Pitt Bacteremia score	Alive, n (%)	Died, n (%)	Total, n (%)	Mortality stratified by qPitt Bacteremia score
0	45 (33%)	4 (8%)	49 (26%)	8%
1	36 (26%)	5 (10%)	41 (22%)	12%
2	40 (29%)	11 (22%)	51 (27%)	22%
3	14 (10%)	12 (24%)	26 (14%)	46%
4	1 (1%)	14 (27%)	15 (8%)	93%
5	0 (0%)	5 (10%)	5 (3%)	100%
	136 (73%)	51 (27%)	187 (100%)	

## Discussion

BSIs are serious and relatively common infections associated with an unacceptably high mortality rate as demonstrated by Hattori et al [15] and as evidenced by the findings of the current study. In the present study, 51 of the 187 patients with BSI died during their hospitalization resulting in an all-cause in-hospital mortality of 27.3% in this cohort. This is largely consistent with the reported mortality outcomes of BSI in the literature [16]. Furthermore, the present study observed that patients with BSI who died during their hospitalization tend to be older than patients who were alive at the time of discharge suggesting that age has a significant impact on the outcome of BSI. This observation may be explained by the association of aging with increased comorbidities and immunosenescence [17, 18].

Gram-negative sepsis with *E. coli*, *Klebsiella pneumoniae*, *Pseudomonas aeruginosa*, and *Acinetobacter baumannii *occurred in two-thirds of the patients in this study. Conversely, BSI from Gram-positive isolates including *S. aureus* and Enterococcus species accounted for the remaining one third of the patients examined. This dominance of Gram-negative bacteria is consistent with the local epidemiology [19].

Numerous scoring methods have been developed to evaluate the severity of sepsis and to predict mortality, some of which are complex and at times cumbersome for routine use [20]. Contrarily, the C-reactive protein/albumin ratio and qPitt bacteremia score [10, 21] are two relatively simple to use, widely available, and readily obtained severity scores in patients with sepsis. Hence, we herein examined the relationship between these two scores and mortality among hospitalized patients with sepsis due to BSI.

Higher CRP levels have been shown to indicate a more severe disease status and a worse prognosis in patients with sepsis [22]. Similarly, serum albumin concentration has recently been postulated as a predictive biomarker of mortality in sepsis [23]. We found that deceased patients with sepsis secondary to BSI have significantly elevated levels of CRP (mean CRP: 199 mg/L) within 24 hours of blood culture collection. Likewise, serum albumin levels were much lower (mean serum albumin: 25 g/L) in the patients who died compared to those who survived. Despite the above mentioned, the prognostic role of either markers alone in patients with BSI is currently limited. It has been reported that the CRP/albumin ratio might serve as a marker of clinical outcome instead [24]. Nevertheless, the role of the CRP/albumin ratio in predicting mortality in patients with BSI is currently unclear hence we examined this further in the present study. We demonstrated that deceased patients with BSI had a significantly higher mean CRP/albumin ratio (8.6 ±1.7) when compared to those who survived (5.3 ±0.8). This finding is consistent with a recent systematic review and meta-analysis [25].

The Pitt bacteremia score has been in use for the past three decades to stratify disease severity in patients with BSI [12]. A recently simplified easy-to-use version of the Pitt bacteremia score, referred to as the qPitt bacteremia score was developed [26]. This new tool uses binary variables for body temperature, blood pressure, respiratory rate, cardiac arrest event, and mental status [26].

Since the use of the qPitt bacteremia score in Gram-negative BSI is limited and its relationship with CRP/albumin ratio in patients with BSI is largely unexamined, we sought to explore this further. In this study where two-thirds of the BSI isolates were Gram-negative bacilli, a higher qPitt bacteremia score (2.8 ±0.4) was demonstrated to be significantly associated with an elevated all-cause in-hospital mortality. Moreover, we demonstrated a consistent and incremental increase in all-cause in-hospital mortality rates with higher qPitt bacteremia scores suggesting its utility as a tool for disease severity in patients with BSI. In this study, a qPitt bacteremia score of ≥3 is shown to be associated with unacceptably high in-hospital mortality in in patients with bacteremic BSI.

The present study has several limitations, in addition to those inherent in its retrospective nature and being a single-center research. First, a relatively small sample size potentially restricts conclusions from this research. Second, heterogeneity of the blood culture isolates. Third, factors known to impact mortality outcomes, such as timing and appropriateness of empirical antibiotics and sources of bacteremia and their control were not studied. Despite these limitations, this study had a clearly defined study population, simultaneously examined the relationship between CRP, albumin, CRP/albumin ratio, EWS, and the qPitt bacteremia score with short-term mortality in bacteremic patients and explored the utility of Quick Pitt bacteremia score in BSI resulting from heterogenous blood culture isolates with a predominance of Gram-negative pathogens. We believe that the findings of this study will add to the existing knowledge and will likely impact local clinical practice.

## Conclusions

Hospitalized adult patients with bacteremic BSIs have high mortality. Deceased patients with sepsis secondary to BSI have significantly elevated levels of CRP, lower serum albumin concentrations, and significantly higher mean CRP/albumin ratio. Furthermore, a higher qPitt bacteremia score is significantly associated with an elevated all-cause in-hospital mortality with a score of ≥3 is associated with unacceptably high mortality in patients with bacteremic BSI. We believe that the findings of this research may inform clinical interventions and improve clinical outcomes of patients with BSIs.
